# Delayed Presentation of Traumatic Diaphragmatic Hernia: a Diagnosis of Suspicion with Increased Morbidity and Mortality

**DOI:** 10.5812/traumamon.7125

**Published:** 2013-05-26

**Authors:** Farooq Ahmad Ganie, Hafeezulla Lone, Ghulam Nabi Lone, Mohd Lateef Wani, Shabir Ahmad Ganie, Nasir-u-din Wani, Masaratul Gani

**Affiliations:** 1 Department of Cardiovascular and Thoracic Surgery, SKIMS Soura, Srinagar, India; 2 Department of General Surgery, Kidney Hospital, Srinagar, India; 3 Department of J and K Health Services, University of Kashmir, Kashmir, India

**Keywords:** Hernia, Diaphragmatic, Traumatic, Wounds, Nonpenetrating, Delayed Diagnosis

## Abstract

**Background:**

Diaphragmatic rupture due to blunt or penetrating injury may be a missed diagnosis in an acute setting and can present with a delayed complication with significantly increased morbidity and mortality.

**Objectives:**

The objective of this study is to better understand why diaphragmatic tears with delayed presentation and diagnosis are so often missed and why traumatic diaphragmatic tears are difficult to diagnose in emergency settings and how they present with grievous complications.

**Patients and Methods:**

Eleven patients with diaphragmatic hernias with delayed presentation and delayed diagnosis were operated within the last five years. All patients presented with different complications like gut gangrene or respiratory distress.

**Results:**

Out of eleven patients who were operated on for diaphragmatic hernia, three patients (27%) died. Three patients required colonic resection, one patient needed gastrectomy and one patient underwent esophagogastrectomy.

**Conclusions:**

A small diaphragmatic tear due to blunt trauma to the abdomen is difficult to diagnosis in acute settings due to ragged margins and possibly no herniated contents and usually present with a delayed complication. Therefore a careful examination of the entire traumatized area is the best approach in treating delayed presentation of traumatic diaphragmatic hernia prior to development of grievous complications.

## 1. Background

Diaphragmatic injuries resulting from either blunt or penetrating trauma are relatively rare. The well-known symptom of blunt trauma includes a sudden increase in intra-abdominal pressure, especially during vehicle collision, which tears a weak point in the diaphragm. Small diaphragmatic hernias are often not diagnosed until months or years later when the patients become symptomatic. Knowledge of diaphragmatic hernia is essential for both physicians and surgeons in atypical abdominal and respiratory discomfort, especially when there is history of trauma. Diaphragmatic rupture occurs due to blunt or penetrating injury, having either an acute presentation as a part of multi-organ injury or delayed presentation as respiratory distress or gut obstruction (1-3). It can be managed through laparotomy or thoracotomy (4) and with minimal access surgery. Traumatic diaphragmatic hernias are uncommon, yet associated with high morbidity or mortality when diagnosed late. The pathophysiologic effects of ruptured diaphragm affect circulation and respiration. This is due to the impaired function of the diaphragm, compression of the lungs, and displacement of the mediastinum with impairment of the venous return to the heart. The patient with a diaphragmatic rupture often presents with breathlessness which is mistaken for bronchopneumonia especially when a history of injury is not forthcoming. The obstructive phase is when the loop herniation is obstructive and the patient develops distension and strangulation. A CT scan of the chest is most commonly performed to diagnose diaphragmatic hernia.

## 2. Objectives

The objective of this study is to better understand why diaphragmatic tears with delayed presentation and diagnoses are so often missed.

## 3. Patients and Methods

This prospective study was performed at the department of cardiovascular and thoracic surgery at Sher-i-Kashmir Institute of Medical Sciences, Soura, Srinagar Kashmir. Eleven patients who had delayed presentation and delayed diagnosis for diaphragmatic hernia operated within the last five years were assessed. All patients presented with different complications. All were admitted through the emergency department. Nine patients were operated as emergency cases while two cases underwent elective surgical intervention following 3 days of stabilization. Nine patients required emergency work-up while two patients were evaluated and received a work up by routine procedure. All patients underwent a CT scan of the chest. Nine patients needed thoracotomy followed by laparotomy while two cases underwent thoracotomy.

## 4. Results

Eleven patients with a diaphragamatic hernia and a history of blunt abdominal trauma in the past 9 to 12 months were operated with nine patients surviving (88%). All cases were males. Nine patients had herniation on the left side, while two had the herniation on right side of the diaphragm. Nine cases with a left side diaphragmatic hernia were above sixty years of age however the other two patients with a right side hernia were eight and ten years-old. Of the patients with a left side hernia, two had an isolated stomach herniation while three other patients had a stomach and large gut as the herniated organs. Two cases had isolated large gut whereas two patients had a stomach and spleen as the herniated organs. On the right side, the liver was the only herniated organ. Two cases with a right side hernia were eight and ten years old and presented to our institute two months following the trauma with respiratory distress. Nine other patients had left sided diaphragmatic hernia which presented 9 to 12 months following the injury. Three patients were wrongly diagnosed as a left side hydropneumothorax and were referred with a chest tube on the left side in the large intestine (in two cases) and stomach (in one case). Three patients had fecal empyema thorax on the left side, two patients presented with dysphagia and four patients presented with intestinal obstruction. Three patients underwent transverse colectomy with divided loop colostomy; however one patient underwent upper partial gastrectomy and one patient underwent esophagogastrectomy. Both right and left diaphragmatic tears were repaired by 10-0 prolene using interrupted horizontal mattress sutures. Three patients with left side hernia died in post-operative stage and one of them underwent re-exploration twice for anastomotic leak following upper partial gastrectomy with esophagectomy. One patient who died had significantly friable diaphragm and recurrent diaphragmatic hernia on the 1st post-operative day due to disrupted corner sutures. This patient was on a mechanical ventilator for two days before his death. Complete lung expansion following diaphragmatic repair took more than 7 days in the majority of the patients with left side hernia. Patients with right sided hernia repair were discharged on the 5th post-operative day. Three patients with colonic herniation had fecal pneumothorax and one patient had gastric perforation in the herniated area.

## 5. Discussion

Herniation of abdominal contents with gangrene of gut and fecopneumothorax occur relatively early, however, tension hydropnemothorax may present as a late complication of traumatic diaphragmatic tear (5-8). Traumatic diaphragmatic rupture or hernia occurs in 5% with blunt abdominal trauma due to motor vehicle accidents (9). Furthermore Turhan K et al. and Tsuboi et al. report that traumatic diaphragmatic hernias (TDH) are observed in 10% of diaphragmatic injuries, which include blunt trauma, penetrating trauma (firearm injuries and stab wounds) and iatrogenic lesions (10, 11). It is reported that missed diaphragmatic injuries in conservatively-managed patients range from 12% to 66% (12, 13). It is not feasible to estimate the occurrence of diaphragmatic hernia due to blunt abdominal trauma as a significant number of patients manifest late and some are diagnosed while being evaluated for other pathologies. A search of the literature revealed nine cases with colon perforation resulting in tension fecopnemothrax between 1986 and 1997 (8, 9, 14-16). We had two patients with colonic perforation; however, it was not clear whether it was due to gangrene of the large intestine or due to wrongly placed intercostal thoracostomy tube for wrongly diagnosed hydropneumothorax. Immediate surgical intervention may be considered as the gold standard for treating delayed diaphragmatic rupture (17). Patients with herniated gut via left hemi-diaphragm were operated immediately due to complicated presentation while patients with herniated liver on the right side received elective surgery. The diaphragmatic injury can be diagnosed at laparotomy however the preoperative diagnosis is difficult in the absence of simultaneous organ injury (18). We operated on five patients (not included in the study) with small diaphragmatic tears at the initial presentation only because those patients were operated for splenic injury and diaphragmatic tears were accidental findings. The preoperative diagnosis of traumatic diaphragmatic tears are difficult and therefore the actual incidence is unknown. It remains silent for hours to years following injury unless complications develop (19). There are usually no clinical symptoms to diagnose an isolated blunt diaphragmatic injury, and it is usually diagnosed during examination for other injuries (18). Missed diaphragmatic injuries result inevitably in intra-thoracic herniation due to the intra-abdominal to intra-thoracic pressure gradient reaching up to 100 mmHg during Valsalva maneuver (normal: 2 – 10 mmHg) (20). Although difficult to detect, trauma surgeons should pay attention to diagnose diaphragmatic tears upon initial admission because a delay in diagnosis increases mortality from 3% to 30% (21, 22). In our study, we had a mortality rate of 27% in this group of patients. All those patients who survived had significant in-hospital morbidity,except two patients with right side diaphragmatic hernia. Murray et al. (23) reported that 62% of the patients with occult diaphragmatic injuries have a normal chest radiograph. Miller et al.(24) and Demetriades et al.(22) reported this to be 43% and 11% respectively. A herniation at the costophrenic angle may be misdiagnosed as pleural effusion or haemothorax on the chest radiograph and a chest tube could accidentally be placed into the herniated organs (25). Sensitivity of conventional CT scan ranges between 14% to 82% while specificity ranges between 76% to 100% (26).

Five patients in our series who had a CT scan of the chest at the time of initial admission which did not reveal any defined diaphragmatic tear or herniation of intra-abdominal contents. Patients who were diagnosed with diaphragmatic hernia at initial admission but not included in this study had established herniation at initial presentation with intra-operative finding of a diaphragmatic tear of more than 4cms. Their chest X-ray as well as chest CT scan was diagnostic of herniation in the respective clinical settings. It was seen that a conventional CT scan was not useful in determining an acute diaphragmatic tear, however, it was a beneficial method in detecting the diaphragmatic hernia in the traumatic diaphragmatic hernia group (19). Tracing former records of our patients revealed that all their investigations at the time of trauma were not suggestive of any diaphragmatic tear without herniation although none of the patients underwent MRI of the chest or abdomen.

Traumatic diaphragmatic tears are difficult to diagnose in emergency settings with available diagnostic tools unless it is accompanied by herniation of intra-abdominal contents. Incorrect interpretation of the chest X-ray or only intermittent hernial symptoms are frequent reasons for incorrect diagnosis (27). In our cases initial chest X-rays were normal but on follow-up patients presented with vague symptoms which could not be related to diaphragmatic hernia. Diaphragmatic injury should be suspected in all patients with penetrating as well as blunt injury of the chest and abdomen (28). The majority of complications occurred between one and four years following the injury (29). Almost 88% of the patients presented with complications between 9 and 12 months. Left sided injuries occurred in 68.5% of the patients, 24.2% had right-sided rupture (30), and 1.5% had bilateral rupture. The most commonly herniated organs on the left side are the stomach (80%), omentum, small intestine, colon, and spleen (31). Eighty-eight percent of the cases in our series were diagnoses on the left side.

The literature indicates that plain chest X-ray is diagnostic in 73% of patients with diaphragmatic hernia. The signs of diaphragmatic injury on plain radiographs are intra-thoracic herniation of abdominal viscera, marked elevation of the hemi-diaphragm, distortion of the diaphragmatic margin and contra-lateral mediastinal shift (32, 33). These classic signs are only seen when there is established herniation, otherwise diaphragmatic tear without herniation in acute settings is difficult to diagnose. Ultrasonography can also be diagnostic in patients with traumatic diaphragmatic hernia because it allows defining absent diaphragmatic movements, herniation of viscera or flaps of ruptured diaphragm (2). Ultrasound has been beneficial only when there is established gut herniation whether in an acute setting or in a delayed setting; an isolated diaphragmatic tear was never diagnosed on ultrasound. According to the literature, the surgical approach depends on the departments that manage the patient. General surgeons use laparotomy in 92% (30) and thoracic surgeons carry out thoracotomy in 78% of the cases (34). Usually, laparotomy seems to be more appropriate especially when associated intra-abdominal lesions are suspected (35). The repair of the hernial defect can be made with non-absorbable or absorbable suturing material; however, the use of non-absorbable suturing is widely recommended (36). Both interrupted and continuous techniques are equally effective (37) while simple suture is sufficient in smaller defects, larger defects need a synthetic mesh (30).In our series three patients were operated jointly by thoracic and general surgeons; Two patients underwent colonic resection with divided loop colostomy and one patient required upper partial gastrectomy with splenectomy. All other patients with left side hernia were operated by thoracic surgeons and the approach was thoraco-abdominal. Small laparotomy was performed to look for viability of reduced contents, constriction band around the herniated area and any volvolus of the reduced contents. One patient who required upper partial gastrectomy with lower esophagectomy had anastomotic leak twice and died due to sepsis with renal shutdown. We used 10 prolene suture with interrupted horizontal mattress technique for closing the defect in diaphragm. None of the patients needed prolene mesh to close off the defect. Almost ninety percent of the diaphragmatic ruptures occurred on the left hemi-diaphragm, primarily because of the protective effect of the liver on the right hemi-diaphragm and possibly because of the under-diagnosis of the right-sided injuries (38). Nine out of eleven patients with delayed presentation were on left side in our study. Strangulation of herniated intestines should be considered when there is radiologic evidence of herniation (39). Strangulation of the intestine at the hernia site might be associated with distension with chest X-ray resembling hydropneumothorax which was the finding in our three patients. The “interval” phase of herniation, defined as occult diaphragmatic herniation, is often asymptomatic or manifests as vague dyspeptic symptoms or upper abdominal discomfort (40). Visualization of the diaphragm is difficult because of its thinness, its dome-like contour, and its continuity with the soft tissues of the abdomen (41). All cases of fecal pneumothorax resulting from diaphragmatic herniation and perforation of the colon share a uniform history of a stab wound to the chest (8). Thoracotomy with reduction of herniated organs can be performed safely with satisfactory results (42). Isolated thoracotomy has never been a better approach when there is delayed presentation of diaphragmatic hernia, intestinal or mesentry constriction at hernia site required to be assessed properly following reduction; therefore, thoraco-abdominal approach was the best approach. The role of thoracoscopy and barium meal remains vital in diagnosis of diaphragmatic hernia; however because of the non-availability of thoracoscopy in our center we did not perform this.

A small diaphragmatic tear is very challenging to diagnose in patients with blunt trauma to the abdomen in acute settings due to ragged margins of injured diaphragm and the lack of herniated contents in an acute setting. There is slow herniation of the gut which can manifest variably. Search for diaphragmatic injury is the best approach when dealing with a delayed presentation prior to it presenting with another emergency significantly increasing morbidity and mortality. Elderly patients are at the highest risk of diaphragmatic tear due to blunt trauma; possibly due to a weakened diaphragm. The thoraco-abdominal approach gives maximum flexibility in dealing with delayed and complicated diaphragmatic hernia. It is concluded that with present diagnostic armamentarium few cases of diaphragmatic tears may be missed.
